# The distinct roles of IL-37 and IL-38 in non-small cell lung carcinoma and their clinical implications

**DOI:** 10.3389/fimmu.2025.1564357

**Published:** 2025-03-21

**Authors:** Jiwei Zhang, Steven G. Wise, Shunqing Zuo, Shisan Bao, Xufeng Zhang

**Affiliations:** ^1^ Department of Thoracic Surgery, Songjiang Hospital Affiliated to Shanghai Jiao Tong University, Shanghai, China; ^2^ School of Medical Sciences, Faculty of Medicine and Health, The University of Sydney, Sydney, NS, Australia

**Keywords:** NSCLC, IL-37, IL-38, differential, precision medicine

## Abstract

Lung cancer, a significant global health challenge, is primarily classified into non-small cell lung cancer (NSCLC) and small cell lung cancer. Despite advancements in targeted therapies and immunotherapies, NSCLC outcomes remain poor, with low five-year survival rates. Given the lung’s constant exposure to the environment and the presence of mucosal-associated lymphoid tissues, immunity plays a crucial role in NSCLC development. Immune checkpoint inhibitors (ICIs) targeting PD-1/PD-L1 have shown promise. However, adverse immune events limit their efficacy. This review highlights the contrasting roles of IL-37 and IL-38 in NSCLC pathogenesis. IL-37, an anti-inflammatory cytokine, suppresses tumour growth. It achieves this by modulating macrophage polarization and dendritic cell maturation. Correlations between intra-tumoral IL-37 expression and improved survival suggest a protective role in NSCLC. This may be mediated through VEGF inhibition and immune regulation. Conversely, IL-38, while anti-inflammatory in certain contexts, exhibits a pro-tumorigenic role in NSCLC. IL-38 enhances tumour progression by increasing pro-inflammatory cytokine secretion and facilitating immune evasion, potentially through NF-κB signalling. Notably, IL-38 negatively regulates IL-37, further promoting tumorigenesis. Emerging data suggest that IL-37 has therapeutic potential in inhibiting NSCLC metastasis and supporting immune modulation. In contrast, IL-38 presents a potential target for mitigating pro-inflammatory microenvironment effects. The distinct roles of these cytokines emphasize the complex immune dynamics in NSCLC. Further exploration of their molecular mechanisms and therapeutic implications is warranted. Targeting IL-37 and IL-38 may offer novel strategies for enhancing NSCLC treatment outcomes

## Lung cancer

Lung cancer remains a significant public health concern. Despite advancements in genetic diagnosis, immunotherapy, and other treatments, including targeted therapies, NSCLC outcomes often remain poor. NSCLC accounts for 85% of lung cancers and is further classified into three major histopathological subtypes: adenocarcinoma, squamous cell carcinoma, and large cell carcinoma (1).

Given the lung’s association with mucosal-associated lymphoid tissues, both humoral and cellular immunity play vital roles in its protection. The lung’s epithelial surface is constantly exposed to the environment, making it susceptible to various microbial challenges (2). Carcinoma tissues often exhibit substantial leukocyte infiltration. This is accompanied by increased expression of pro- and anti-inflammatory cytokines within the tumour microenvironment, aiming to inhibit tumour growth.

Immune checkpoint inhibitors (ICIs), such as those targeting PD-1/PD-L1 or CTLA-4 (3), represent a class of immunotherapy drugs that prevent tumour evasion of host tumour antigen-specific T cell immunity. These therapies have shown promising outcomes in various malignancies. However, some NSCLC patients experience immune-related adverse events, including severe diarrhea, colitis, dermatitis, hepatic damage, or thyroiditis, following PD-1/PD-L1 treatment (4) Managing these adverse effects can be challenging and may involve immunosuppressives, steroids, and withdrawal of ICI therapy. Unfortunately, some patients still experience fatal outcomes, highlighting the intricate nature of host immunity in immuno-oncology and necessitating further research.

This mini-review specifically focuses on the distinct roles of IL-37 and IL-38 in the context of NSCLC development.

### IL-37

IL-37 expression has been identified in diverse tissues, including lymph nodes, thymus, lung, intestine, uterus, and various immune cell types (NK cells, activated B cells, monocytes), as well as epithelial cells such as keratinocytes and others (5). Primarily recognized as an anti-inflammatory cytokine, IL-37 demonstrates the ability to suppress both innate and adaptive immunity (6), leading to an overall reduction in the host’s immune response, including its potential impact on tumorigenesis.

The anti-inflammatory functions of IL-37 are attributed to its ability to hinder dendritic cell maturation (7) and modulate macrophage polarization. This modulation promotes the M1 macrophage phenotype while inhibiting the M2 subtype (8).

Dysregulation of IL-37 has been observed in various autoimmune diseases, such as psoriasis, Graves’ disease, systemic lupus erythematosus, ulcerative colitis, and Crohn’s disease (9). In these conditions, IL-37 may play a role in inhibiting pro-inflammatory responses.

### IL-38

IL-38, a member of the IL-1 superfamily, is constitutively expressed in various tissues, including the heart, lung, intestine, urogenital system, and skin (10). Its primary function is to maintain homeostasis within the tissue microenvironment by inhibiting or suppressing inflammatory responses (11).

One mechanism of IL-38’s anti-inflammatory action involves its release from apoptotic cells, which limits inflammatory macrophage responses (12). In a humanized allergic asthma NOD/SCID murine model, IL-38 demonstrated anti-inflammatory effects by inhibiting pro-inflammatory cytokines such as IL-6, TNF, CCL5, and CXCL10. This likely occurs through the modulation of classical signalling pathways, including STAT1, STAT3, p38 MAPK, ERK1/2, and NF-κB (13).

Similar to the IL-1α/β receptor antagonist and IL-1R1, IL-38 mediates its anti-inflammatory activities. It plays a crucial role in maintaining homeostasis by balancing the pro- and anti-inflammatory microenvironment (14). Dysregulation of IL-38 can disrupt this balance, leading to an imbalance in the pro- and anti-inflammatory microenvironment and potentially initiating host immunity and the development of inflammatory diseases.

IL-38 expression is upregulated in inflamed skin (15) and actively inflamed tissues of inflammatory bowel disease, indicating its anti-inflammatory role in response to focal inflammation. However, in psoriatic skin, IL-38 expression is downregulated in response to stimulation by pro-inflammatory cytokines such as IL-36γ, IL-17, and IL-22. This suggests that IL-38 counteracts the biological processes induced by pro-inflammatory cytokines in epithelial and endothelial cells, thereby attenuating the severity of autoimmunity.

This review aims to emphasize the evolving connection between IL-37 and IL-38 expression and NSCLC development, with a particular focus on their potential clinical implications.

### IL-37 and NSCLC

IL-37 is constitutively expressed in the cytoplasm of alveolar epithelial cells in the lungs of healthy individuals. In NSCLC patients, intra-tumoral IL-37 expression is significantly suppressed at both the mRNA and protein levels (16). This suggests a potential disruption of IL-37 function within the lung microenvironment of NSCLC patients. The precise cause and effect relationship between this suppressed IL-37 expression and dysregulated host immunity remains to be fully elucidated.

Importantly, a correlation has been observed between intra-tumoral IL-37 expression and the overall survival of NSCLC patients (16). This suggests a potential protective role for IL-37 in NSCLC development. This finding aligns with observations in colorectal cancer patients, where a positive correlation exists between intra-tumoral IL-37 expression and overall survival/disease-free period (16). Furthermore, an inverse correlation has been reported between intra-tumoral IL-37 expression and the depth of invasion of NSCLC (16), as well as the distance of metastasis (16). These findings are supported by evidence indicating that the intracellular mature form of IL-37 suppresses NSCLC metastasis via the Rac1 signalling pathway. This is consistent with observations in hepatocellular carcinoma (17).

Collectively, these data suggest that IL-37 may play a beneficial role in inhibiting NSCLC development.

From a mechanistic perspective, an animal model of lung cancer has been employed to explore the impact of IL-37 on tumour growth. Reduced tumour size was observed in nude mice recipients of IL-37 transgenic human lung cancer cells compared to those with mock transgenic cells. This reduction occurred in a time-dependent manner (16). The successful expression of transgenic IL-37 was confirmed at both mRNA and protein levels (16). The diminished tumour size correlated with a decrease in VEGF and subsequent neovascularization in the tumours of recipients receiving IL-37-transfected cells. No significant impact on infiltrating leukocytes was observed (16). IL-37 suppresses the invasion and migration of NSCLC cells *in vitro* via the IL-6/STAT3 signalling pathway (18). These findings shed light on how IL-37 may inhibit the development of NSCLC *in vivo*, considering that VEGF and neovascularization are recognised as pivotal factors in NSCLC development and are current targets for clinical intervention.

It is worth noting that the absence of infiltrating leukocytes in the inoculated tumours among recipients with IL-37 transgenic and non-transgenic cancer cells. This may be attributed to the use of nude mice in this study. Nude mice lack immune cells, which would normally prevent the rejection of injected human cells from rodent hosts. However, it is well-established that host cellular and humoral immunity play crucial roles in NSCLC development (19). Future studies should build on these initial findings by confirming the role of IL-37 in humanised animal models, particularly investigating the interplay of different cells and cytokines in response to the challenge from human NSCLC cells *in vivo*.

**Figure 1:**
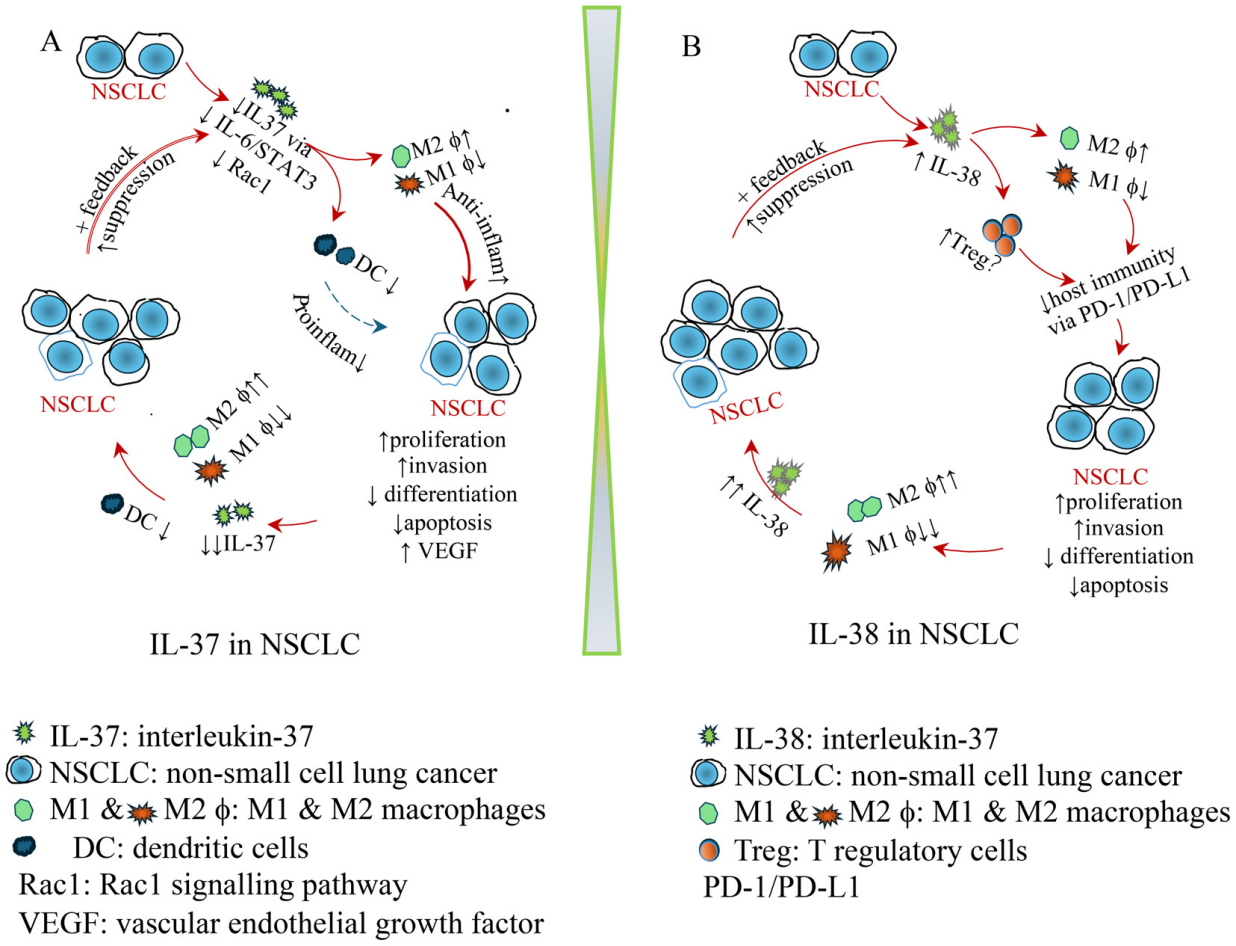
Schematic diagram illustrating the role of IL-37 and IL-38 in NSCLC. **(A)** IL-37 in NSCLC: protective role of IL-37. Decreased intra-tumoral IL-37 leads to a reduction in dendritic cells (DCs) and M1 macrophages, while increasing M2 macrophages. Consequently, anti-inflammatory cytokines are elevated, whereas pro-inflammatory cytokines are reduced, promoting NSCLC proliferation, invasion, and neovascularization, while decreasing differentiation and apoptosis. This contributes to a pro-tumoral environment. As NSCLC continues to grow, IL-37 suppression intensifies, leading to a further reduction in DCs and M1 macrophages. This, in turn, accelerates NSCLC progression, creating a feedback loop that further drives tumour development. **(B)** IL-38 in NSCLC. Upregulated intra-tumoral IL-38 contributes to a reduction in dendritic cells (DCs) and M1 macrophages while increasing M2 macrophages and regulating Treg cells. This leads to compromised host immunity via the PD-1/PD-L1 pathway, promoting NSCLC development, proliferation, invasion, and neovascularization, while decreasing differentiation and apoptosis. As NSCLC continues to grow, IL-38 elevation intensifies, further reducing M1 macrophages while promoting M2 macrophages, leading to even higher IL-38 levels. This, in turn, accelerates NSCLC progression, creating a feedback loop that further drives tumour development.

Circulating IL-37 levels exhibit significant differences between TNM stage I-II and III-IV in NSCLC (18), implying a potential role for IL-37 in inhibiting distant metastasis. The tumour microenvironment plays a critical role in carcinogenesis. Future studies should elucidate how the systemic immune response may synergise or provide supportive roles in suppressing NSCLC development *in vivo* (**Figure 1A**). *In vitro* investigations have demonstrated that exogenous IL-37 inhibits lung cancer cell proliferation and migration (18), while suppressing epithelial-to-mesenchymal transition via the STAT3 signalling pathway. The observed inverse correlation between IL-37 expression in NSCLC and the depth of colorectal cancer invasion (20) suggests that IL-37 may similarly regulate host immunity in the lung and gut, governed by the mucosal lymphoid-associated tissue immunological system. Moreover, IL-37 expression is closely associated with overall disease-free status and survival in both NSCLC and colorectal cancer, further supporting the protective role of IL-37 in the mucosa during the development of malignancies.

However, there is presently no substantial correlation between IL-37 expression and age or sex in NSCLC patients (16). This lack of correlation could be attributed to the relatively small sample size reported to date, encompassing a total cohort of 182 from a single centre. With an average age close to 55 years in this cohort (16), these patients are less likely to derive the protective effects of sex hormones, which typically reduce the incidence of NSCLC in women of fertile age. Comparable findings have been documented in colorectal cancer studies, where age and sex do not emerge as significant prognostic factors (21).

It is widely recognized that smoking constitutes a major risk factor for the development of lung cancer. However, surprisingly, there is presently no significant difference in intra-tumoral IL-37 expression between smokers and non-smokers in NSCLC patients (16). This unexpected observation may be attributed to the limited sample size in the study. It remains plausible that IL-37 may not be intricately involved in smoking-related carcinogenesis of NSCLC, a hypothesis that warrants clarification through larger, multicentre studies in the future.

IL-37 has been shown to impede tumorigenesis in the lung through the Rac1 pathway (22), possibly by suppressing the production of pro-inflammatory cytokines and chemokines (23). This protective function of IL-37 in tumours is not exclusive to lung cancer but extends to various other cancers, such as human hepatocellular carcinoma (24), where it may inhibit the polarization of M1 macrophages and/or angiogenesis in animal models. Evidence from hepatocellular carcinoma indicates that elevated IL-37 levels and the presence of infiltrating CD1a^+^ dendritic cells are associated with higher overall survival rates (25). The increased dendritic cells may enhance professional antigen presentation, potentially influencing the differential polarization of macrophages (25). This could shed light on the diverse roles played by macrophages in malignancy development, suggesting distinct mechanisms of carcinogenesis or microenvironments. The polarization of M0 macrophages into M1 or M2 tumour-associated macrophages plays distinct roles in the initiation and development of malignancy, with M1 macrophages generally exhibiting anti-tumour functions and M2 macrophages promoting tumour progression (26). This topic, however, will not be discussed further in this mini-review.

Additionally, IL-37 is largely produced by monocytes/macrophages and dendritic cells (27). IL-37 promotes host anti-tumour immunity by enhancing the recruitment of dendritic cells in hepatocellular carcinoma (25). It is reported that tendritic cell maturation is compromised in NSCLC biopsies. Moreover, the poor antigen-presenting function of tumour-infiltrating DCs, even after TLR stimulation, and the impaired migratory response of both tumour-infiltrating mDCs and pDCs towards CCL21 and SDF-1 (28). These findings suggest that DCs play a crucial role in immune surveillance during NSCLC development.

It is worth noting that the quantification of IL-37 immunohistochemistry in the studies utilized a subjective, semi-quantitative methodology based on visualization alone (16), which may compromise objective evaluation. Ideally, a more accurate and objective approach would involve computerized automated quantification (21).

Moreover, the precise role of IL-37 during the development of NSCLC remains to be clarified. Studies should be designed to explore its potential therapeutic value by genetically manipulating IL-37 in animals, particularly in humanised mice (29). This approach would allow us to better understand the interaction between IL-37 and NSCLC, particularly in immunocompetent hosts, and advance the development of precision medicine.

Furthermore, intrahepatic IL-37 expression is significantly reduced in HCC tissue (24), compared to that of non-HCC tissues. Its expression correlates with overall survival and disease-free survival, suggesting that IL-37 plays a protective role in HCC development. More specifically, a positive correlation exists between intrahepatic IL-37 levels and infiltrating CD57^+^ NK cells within HCC tissues, consistent with *in vitro* investigations showing that exogenous IL-37 promotes the recruitment of CD57^+^ NK cells (24). Importantly, this observation is further validated in IL-37 transgenic HCC models, showing significantly reduced HCC growth accompanied by an increased presence of CD57^+^ NK cells (24). This suggests that IL-37 may contribute to tumour suppression by enhancing NK cell recruitment.

Similarly, colonic IL-37 expression is also significantly reduced in colorectal cancer tissues, possibly due to its role in neutrophil recruitment (20). Moreover, IL-37 expression correlates with cancer invasion and prognosis, further supporting its involvement in tumorigenesis.

### IL-38 and NSCLC

In contrast to IL-37, intra-tumoral IL-38 expression in NSCLC demonstrates a significant inverse correlation with both disease-free and overall survival in Japan (30), implying that IL-38 contributes to the development of NSCLC. This correlation aligns with the significant differences observed in intra-tumoral IL-38 levels between well- and poorly differentiated NSCLC, indicating that higher IL-38 levels are associated with poorer differentiation (30). The researchers suggest a possible underlying mechanism for the inverse correlation between intra-tumoral IL-38 expression and overall survival: elevated IL-38 in NSCLC patients may inhibit IL-36, possibly suppressing pro-inflammatory responses and promoting NSCLC progression. Furthermore, IL-38 expression exhibits significant variations in relation to the depth of NSCLC invasion, lymph node invasion, and disease stages, reinforcing the role of IL-38 in promoting the development of NSCLC (30). It is noteworthy that substantial differences in IL-38 expression exist between NSCLC patients with and without pleural invasion or with and without vascular invasion, underscoring that IL-38 not only contributes to local progression but also facilitates distant invasion in NSCLC. Additionally, it is noted that the quantification of intra-tumoral IL-38 was conducted in an eyeball manner (30), which should use more objective automated image analysis system (31–33).

Moreover, to investigate the possible mechanism, IL-38-plasmid-transfected Lewis lung carcinoma cells were adoptively transplanted subcutaneously into immunocompetent syngeneic mice (34). Tumours with IL-38 transfection grew significantly larger than non-IL-38-transfected Lewis lung carcinoma cells. Infiltrating CD3+ and CD8+ T cells were significantly reduced in IL-38-transfected tumours, suggesting that IL-38 inhibits the recruitment of CD8+ T cells during lung cancer development. This was further confirmed by CD8+ depletion, which eliminated the difference in tumour growth (34).

At the molecular level, pro-inflammatory cytokines (IFN-γ, TNF, IL-17A) were significantly reduced in IL-38-transfected tumours, further supporting the anti-inflammatory function of IL-38. Additionally, IL-38 preferentially promoted M2 macrophage function rather than M1, which in turn facilitated lung cancer progression. Furthermore, IL-38 released from apoptotic cells inhibited IL-6 production by attenuating the JNK/AP-1 pathway, thereby modulating macrophage responses (12), which could influence NSCLC development.

However, these findings suggest that IL-38 may play a more complex role in NSCLC through multiple signalling pathways. This complexity should be considered in the development of precision medicine strategies.

Then, ICIs (Immune checkpoint inhibitors) have seen increasing use in NSCLC patients with promising results (35). A positive correlation has been identified between IL-38 and PD-1/PD-L1, suggesting that NSCLC patients with high IL-38 expression exhibit a higher TNM score and shorter disease-free survival, particularly in PD-L1-negative tumours where T cell activity is presumed to be less suppressed. Because it has been reported that the outcomes of anti-PD-1 therapy are also significantly influenced by age and sex (i.e., younger and male NSCLC patients have much better overall survival than older females), there is no evidence from this single study (36), and further investigation is needed in future research.

This observation implies that IL-38 may contribute to the progression of NSCLC, possibly through a negative association with PD-1/PD-L1 molecules. IL-38 has been shown to increase the secretion of various pro-inflammatory cytokines, including IL-6, CCL5, and CXCL10, which enhance tumour cell proliferation and migration. Elevated IL-38 expression correlates with the expansion of the pro-inflammatory microenvironment in tumours, potentially contributing to NSCLC progression.

There is currently no direct evidence linking IL-38, Treg cells, and host immunity in NSCLC. However, findings from NSCLC research suggest that IL-38 may promote tumour development by regulating the polarization of infiltrating macrophages in cancer tissues, referred to as tumour-associated macrophages (TAMs) (37). TAMs are divided into two subsets based on their surface markers and functions: classically activated M1 macrophages and alternatively activated M2 macrophages (26). M1 TAMs typically exhibit anti-tumour functions, including direct cytotoxicity through the release of ROS and NO, as well as antibody-dependent cell-mediated cytotoxicity to kill tumour cells. This aligns with the findings of Kinoshita et al., who observed substantially reduced infiltrating lymphocytes and suppressed pro-inflammatory cytokines (34). However, their study did not identify TAMs, which should be verified in future research. Additionally, it has been reported that IL-38 is released from apoptotic cells to suppress macrophage functions, promoting anti-inflammatory responses (12).

The observations from Yuan et al. indicate that colorectal cancer patients with significantly high IL-38 expression and low PD-1 expression exhibited superior survival outcomes compared to other expression combinations (32). It is, therefore, important to note that the precise mechanisms underlying IL-38’s impact on PD-1/PD-L1 expression in NSCLC remain speculative and warrant further investigation. Genetic manipulation of human NSCLC cells *in vitro* and/or NSCLC animal models *in vivo* would be invaluable in elucidating these mechanisms. Additionally, exploring the correlation between IL-38 expression, PD-1, and FoxP3 in the draining lymph nodes of NSCLC—similar to studies conducted in colorectal cancer patients (32) —could provide deeper insights into the role of IL-38 in NSCLC pathogenesis. Multicentre studies involving diverse racial backgrounds are particularly recommended to enhance these findings. Collectively, these observations suggest that IL-38 plays a pro-tumorigenic role by shifting the immune response toward a pro-inflammatory phenotype, promoting immune evasion, and facilitating the growth and spread of NSCLC (**Figure 1B**).

IL-38, an anti-inflammatory cytokine, plays a critical role in maintaining physiological homeostasis and may also suppress host inflammation in autoimmune diseases. For example, local IL-38 expression is reduced in psoriatic skin (14). IL-38 has multifaceted roles in inflammation and cancer (38). In addition to its previously mentioned function, IL-38 inhibits colorectal cancer development in a DSS-induced colorectal cancer model using IL-38 transgenic mice by suppressing tumour growth and metastasis (39). This suppression of the development of CRC occurs through inhibition of the extracellular signal-regulated kinase (ERK) signalling pathway, thereby promoting cancer cell apoptosis (39). These findings highlight the complexity of IL-38 signalling and its potential dual roles in tumour progression, depending on the tumour microenvironments. The precise underlying mechanisms require further investigation in both animal and human studies.

IL-38 appears to play a protective role in colorectal cancer. This consistent finding is also observed in human colorectal cancer (21), where intra-tumoral IL-38 expression is suppressed. Moreover, colonic IL-38 expression correlates with overall survival in colorectal cancer patients, further supporting its protective function in malignancy. This aligns with findings in NSCLC, where IL-38 expression is also linked to improved patient outcomes.

In contrast, constitutively expressed IL-38 in normal skin is suppressed in human cutaneous squamous cell carcinoma (40). However, in a skin cancer animal model, IL-38 deficiency promotes tumour growth by enhancing cancer cell proliferation and migration *in vivo* and *in vitro*. This is demonstrated using keratinocyte-specific IL-38 knockout mice, where tumour progression is driven *via* the JNK/AP-1 signalling pathway (40).

The clinical implications of IL-38 in NSCLC are currently limited, with few publications in the literature. As mentioned above, more precise studies on the underlying mechanisms of IL-38 in anti-NSCLC therapies should be planned, particularly in relation to PD-1/PD-L1 interactions in clinical samples. These findings should then be verified in both *in vivo* and *in vitro* animal models to support potential therapeutic precision medicine.

There is no firm evidence distinguishing the interaction between IL-37 and IL-38 in NSCLC. However, based on the literature, it is speculated that although both are generally classified as anti-inflammatory cytokines, their distinct roles in NSCLC, as described above, are noteworthy. This discrepancy may be attributed to the dual role of IL-37 observed in different diseases (41), which depends on whether its activation occurs extracellularly or intracellularly, such as in the gastrointestinal mucosa (42).

More specifically, intracellular IL-37 acts as a pro-inflammatory mediator by triggering caspase-1 activation; whereas extracellular IL-37 binds to IL-18R and activates GSK3β, thereby suppressing IL-18’s pro-inflammatory function (41). It is possible that intra-tumoral IL-37 in NSCLC is primarily intracellular and pro-inflammatory, thereby inhibiting tumour growth. In contrast, IL-38 may function as an anti-inflammatory cytokine, promoting NSCLC development by suppressing host inflammation and impairing immune surveillance. This speculation will be verified in the future.

Furthermore, the primary objective of this mini-review is to elucidate the interactions among IL-37/IL-38, lymphocytes, macrophages, dendritic cells, and NSCLC. Additionally, given that we are physician-scientists rather than molecular biologists, we recognise that a more comprehensive investigation of signalling pathways should be conducted in future studies using *in vivo* and *in vitro* NSCLC models.

## Potential translational application

These findings highlight the complex and context-dependent role of IL-38 in cancer. Further research into the molecular mechanisms underlying these effects, including potential interactions with other cytokines and immune cells, is crucial for exploring the potential therapeutic implications of targeting the IL-38 pathway in cancer treatment.

The effect of a neutralising anti-IL-38 antibody has been demonstrated in a mammary carcinoma mouse model. Promising outcomes were observed, particularly when combined with chemotherapy, where it exerts a synergistic effect through γδ T cells (43). This aligns with other studies showing that anti-IL-38 antibody treatment, targeting both human and mouse IL-38 proteins, also exhibits a tumour-inhibitory role in animals receiving epithelial-mesenchymal transition cancer cells (44). To date, no clinical trials have been conducted.

While no reports currently exist on targeting IL-37 and/or IL-38 in human cancer therapy, concerns remain regarding the potential for long-term antibody treatment to lead to unintended immune suppression or autoimmunity. Future studies should focus on developing biological agents with high anti-tumour efficacy while minimising adverse effects.

The potential application of IL-37 and/or IL-38 in precision medicine for NSCLC remains largely speculative at this stage. Possible delivery approaches include recombinant proteins (45), gene therapy (46), and mRNA-based delivery (47). These strategies require rigorous validation in preclinical animal models before progressing to randomised clinical trials. Currently, there is no evidence supporting the clinical viability of IL-37 and/or IL-38 in NSCLC treatment. Potential adverse responses, such as the induction of autoimmunity or allergic reactions, warrant careful consideration.

Moreover, single-cell sequencing and spatial transcriptomics are powerful approaches for exploring the immune microenvironment, particularly in NSCLC (48–50). These techniques could provide deeper insights into the interactions among IL-37, IL-38, M1 *vs* M2 macrophages, dendritic cells, and NSCLC. Future studies should explore this further.

Additionally, the most common subtypes of NSCLC are squamous cell carcinoma, large cell carcinoma, and adenocarcinoma (51). Investigating whether IL-37 and IL-38 exhibit differential expression across these NSCLC subtypes would be highly valuable. However, despite an extensive literature search, no studies have explored this to date. These insights will be addressed in future research to advance precision medicine for NSCLC.”

## Conclusion

IL-37 and IL-38, two cytokines with opposing functions, have been identified as significant contributors to NSCLC development. While IL-37 demonstrates a protective role, suppressing tumour growth and promoting favourable immune responses, IL-38 appears to play a detrimental role, enhancing inflammation and immune evasion. The differential functions of these cytokines highlight the complexity of the immune response in NSCLC and underscore the potential for targeting these molecules in the development of immunotherapies. Further studies are needed to fully understand the molecular mechanisms underlying IL-37 and IL-38’s roles in NSCLC, and to explore their potential as therapeutic targets.
